# Interaction mechanisms between liquid organic matter and solid bitumen

**DOI:** 10.1038/s41598-026-36636-6

**Published:** 2026-01-20

**Authors:** Xiao–Hui Lin, Tian Liang, Yan–Rong Zou, Yuan Wang, Yu Zou

**Affiliations:** 1 Wuxi Research Institute of Petroleum Geology, Petroleum Exploration and Production Research Institute, SINOPEC, Wuxi, 214126 China; 2 State Key Laboratory of Deep Earth Processes and Resources, Guangzhou Institute of Geochemistry, Chinese Academy of Sciences, Guangzhou, 510640 China

**Keywords:** Solid bitumen, Liquid hydrocarbon, Molecular docking, Interaction, Geochemistry, Fossil fuels

## Abstract

As a hydrocarbon–rich byproduct of petroleum systems, natural solid bitumen demonstrates dual dissolution and adsorption functionalities toward liquid hydrocarbons. Elucidating these adsorption mechanisms provides critical insights into hydrocarbon expulsion dynamics during bitumen secondary cracking and informs strategies for fluidity modulation. This molecular–scale investigation systematically examines interfacial binding mechanisms governing bitumen–hydrocarbon interactions. Building upon atomistically resolved models, semi–flexible docking simulations were conducted across hydrocarbon compound classes and thermal maturation stages. Quantitative analysis of binding Gibbs free energy differentials between saturated and aromatic hydrocarbons revealed distinct interaction modalities governing solid–liquid organic interfaces. These interfacial interactions exhibit four governing parameters: hydrocarbon type, molecular weight, methyl group density at organic interfaces, and condensation degree. High molecular weight polycyclic aromatic hydrocarbons with elevated condensation degrees and their derivatives display enhanced binding affinities, contrasting with the weak retention observed for light hydrocarbons, small cycloalkanes, and low–weight aromatic species.

Natural solid bitumen originates from prolonged geological degradation of kerogen, crude oil, and related precursors^1,2^. Through successive post–depositional processes including biological alteration, aqueous dissolution, thermal maturation, oxidative weathering, and reservoir degassing, this secondary organic constituent transforms into high–purity solid matter occupying diverse pore networks and micro–fracture systems^3–5^. As a ubiquitous geological archive, solid bitumen preserves critical records of diagenetic evolution and hydrocarbon migration histories in shale formations Maintaining generative associations with petroleum systems, it has remained an essential exploration proxy for identifying hydrocarbon–bearing reservoirs^6–9^. The material exerts fundamental control over reservoir quality through its determinant role in regulating porosity–permeability architecture and subsequent hydrocarbon migration dynamics^10–12^. Its void–occlusion mechanisms induce progressive reservoir compaction while molecular–scale hydrocarbon fractionation through preferential adsorption processes significantly influences production potential. Recent methodological advancements in petrographic and geochemical characterization have elevated its importance as a multidisciplinary indicator for basin analysis, petroleum system modeling, and resource assessment^13–15^.

Extensive investigations have focused on solid bitumen genesis classification schemes, reservoir quality modifications, and wireline log interpretation methodologies^12,16,17^. Nevertheless, systematic mechanistic investigations into natural solid bitumen’s hydrocarbon adsorption characteristics remain conspicuously absent. Geological analyses reveal that in–situ solid bitumen –formed during progressive thermal restructuring of source rock organic matter demonstrates substantial solvent affinity and molecular sequestration capacities toward liquid hydrocarbons, accompanied by hydrocarbon–class–specific adsorption preferences^18,19^. As a transitional organic phase mediating between petroleum generation and residual carbonization, characterization of bitumen–adsorbed hydrocarbon signatures enables operational optimization for targeted component extraction and recovery enhancement^20–22^. Molecular modeling has emerged as an essential methodology for investigating the structure of solid organic matter. This approach constructs macromolecular models based on experimental data from solid–state NMR spectroscopy and elemental analysis, which provide critical information on elemental composition and functional group distribution^23,24^. Such methodology is widely employed to explore the physicochemical properties and adsorption behavior of solid organic materials^25^. Molecular docking, a theoretical simulation method rooted in bioinformatics, predicts the binding pose and binding affinity between molecules by positioning a ligand into the binding site of a receptor based on geometric complementarity and interaction energy calculations^26^. Initially developed for pharmaceutical research, it has been extensively applied to screen lead compounds, elucidate mechanisms of action, and establish structure–activity relationships through the simulation of ligand–target protein interactions^27,28^. In recent years, its applications have expanded into environmental science, materials science, and geochemistry, with growing emphasis on studying organic interactions at solid–liquid interfaces^29^. In geological and geochemical research, molecular docking enables quantitative assessment of the adsorption or binding capacity of different organic matters or liquid hydrocarbons on solid surfaces by calculating the Gibbs free energy (ΔG) difference of the solid–liquid interface system^30^. Combined with molecular dynamics simulations, these atomic–scale computational methods provide the ability to visualize and quantify microscopic solid–liquid interfacial processes^31^. These techniques contribute to a more systematic understanding of fundamental hydrocarbon generation mechanisms and pathways, providing critical insights into the processes of formation, occurrence, and migration of hydrocarbons.

We hypothesize that as the thermal maturity of solid bitumen increases, its macromolecular structure becomes progressively more condensed and aromatic, leading to a gradual reduction in its adsorption capacity for liquid hydrocarbons. Additionally, we propose that hydrocarbons with higher molecular weight are less readily adsorbed by solid bitumen. This hypothesis would be falsified if molecular docking simulations show no significant decrease in adsorption capacity with increasing maturity of solid bitumen, or if higher molecular weight hydrocarbons do not exhibit clearly reduced adsorption affinity compared to lower molecular weight counterparts. To test this hypothesis, this study integrates thermal maturation experiments with macromolecular structural modeling to computationally simulate molecular docking interactions between solid bitumen matrices of varying maturity grades and liquid hydrocarbon species. Using this multidisciplinary approach, we aim to systematically decipher the molecular–scale dynamics at the bitumen–hydrocarbon interface, quantify the adsorption behavior of hydrocarbon compounds onto natural solid bitumen, and establish a theoretical framework for enhancing hydrocarbon production efficiency.

## Samples and experimental methods

### Samples

The Sichuan Basin, a critical petroleum–bearing basin in southwestern China contains abundant hydrocarbon resources. As a principal natural gas production region, northwestern Sichuan exhibits multiple high–quality hydrocarbon source rock formations accompanied by extensive surface oil seepages and solid bitumen veins^32,33^. The analyzed natural solid bitumen specimen (designated HSL) was obtained from the Huoshiling Formation within the Guangyuan region of this basin. The sample exhibits the following fundamental geochemical parameters: total organic carbon (TOC) content of 80.6%, hydrogen index (HI) of 532 mg/g TOC, T_max_ of 439℃, Kerogen type II organic matter.

### Analysis experiment and molecular model construction

This study builds upon the macromolecular model of HSL solid bitumen reported by Liang et al. (2021)^34^. In that study, gold–tube pyrolysis experiments were performed in sealed autoclaves to simulate thermal maturation of HSL solid bitumen.

The sample was crushed to 120 mesh, the inorganic minerals were removed by flotation with zinc bromide solution. Washing repeatedly with deionized water and drying to obtain a pure solid asphalt powder sample. Powdered low–maturity HSL bitumen samples were loaded into gold tubes, purged with argon, sealed by arc–welding, and placed into individual autoclaves. All vessels were connected to a common high–pressure line to maintain consistent yet independently controllable pressure conditions. The assembly was heated in a muffle furnace following a predefined temperature program. Upon reaching target temperatures, selected autoclaves were isolated, removed, and quenched. Solid bitumen residues were collected to represent low–mature, mature, high–mature, and post–mature stages, with corresponding Easy%Ro values (a widely employed geochemical parameter used as an equivalent to vitrinite reflectance for thermal maturity assessment) of 0.74, 1.07, 1.80, 2.65, and 3.02.

The elemental composition (C, H, N, S) of the bitumen samples were determined using an Elementar Vario EL CUBE elemental analyzer. Approximately 5 mg of each sample was sealed in a tin capsule and combusted at 950 °C under an oxygen atmosphere. Under these conditions, carbon, hydrogen, nitrogen, and sulfur were quantitatively converted to CO₂, H₂O, N₂, and SO₂, respectively, with the resulting gases quantified by a thermal conductivity detector (TCD). Oxygen content was measured separately using an Elementar Vario EL III elemental analyzer via high–temperature pyrolysis at 1150 °C, during which oxygen–containing components were converted to CO. The evolved CO was then quantified by TCD to determine the organic oxygen content.

Solid–state ¹³C NMR spectra of the solid bitumen samples at different maturity levels were acquired using a Bruker AVANCE III 400 MHz spectrometer equipped with a 3.2 mm solid–state probe. Spectra were collected using the cross–polarization magic angle spinning (CP/MAS) technique at a spinning speed of 14 kHz with a relaxation delay of 50 s. The obtained NMR spectra were deconvoluted to quantify the relative abundances of distinct carbon functional groups.

**Fig. 1:**
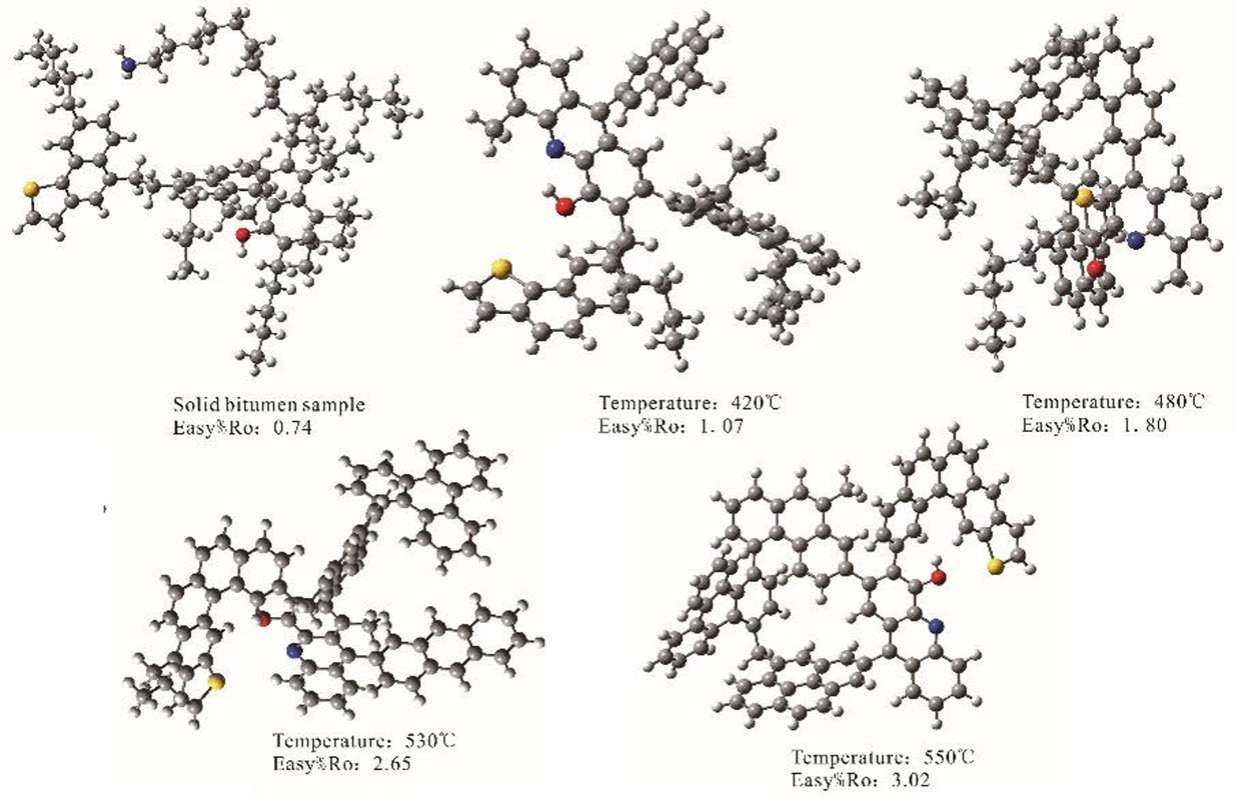
3D molecular structure of the HSL solid bitumen samples.

Molecular modeling was performed using ACD/Labs software (version 2016) based on the elemental composition and relative content of functional groups to construct two–dimensional molecular models representing the carbon–hydrogen skeleton of the bitumen and to simulate the ¹³C NMR spectra of the basic models at low frequency. The simulated NMR signals were broadened to 120 Hz using gNMR (Ivorysoft) to match the experimental NMR spectra. Geometry optimization was carried out using Gaussian 16 at the RB3LYP/3–21G level of theory, converting the 2D models into three–dimensional (3D) structures. Convergence was assessed based on the maximum force, root mean square (RMS) force, maximum displacement, and RMS displacement^35^. GaussianView 6 was used for 3D visualization of the molecular models^36^. A series of representative 3D molecular structure models for HSL bitumen were established, representing the most stable and probable configurations at different maturity stages (Fig. 1).

### Software of molecular Docking

In this study, molecular docking was performed with AutoDock 4.2 to evaluate the ΔG distribution between solid bitumens (at different maturity stages) from the Huoshiling Formation and liquid hydrocarbon molecules, based on pre–constructed molecular structures of HSL solid bitumen. A semi–flexible docking approach was employed, in which the solid bitumen molecules were held rigid in their lowest–energy conformation, while the liquid hydrocarbon molecules remained flexible. The adsorption process was modeled by defining the initial state as separated phases of solid bitumen and liquid hydrocarbons, and the final state as the adsorbed configuration with liquid hydrocarbons on the bitumen surface. The calculated ΔG change serves as a thermodynamic indicator of adsorption spontaneity. More negative ΔG values correspond to enhanced adsorption affinity of liquid hydrocarbons for the solid bitumen surface and increased stability of the formed complexes. This setup is consistent with the sorption behavior of macromolecular organic matter toward mobile compounds. The selected liquid hydrocarbon compounds include: Saturated hydrocarbons (C_2_–C_30_ *n*–alkanes, C_3_–C_10_ cycloalkanes and four different kinds of C_16_ iso–alkanes), and Aromatic hydrocarbons (seven polycyclic aromatic hydrocarbons and five PAH derivatives). Detailed molecular information for these hydrocarbons is provided in Tables 1 and 2. Due to the inherent complexity of liquid hydrocarbons containing numerous known and unidentified components, simulating individual compounds proves computationally prohibitive. Consequently, the compounds were selected not based on their abundance in crude oils or source rocks, but rather to systematically vary in terms of structural class, molecular weight, condensation degree, and methyl group content. This strategic selection enables comprehensive evaluation of how these parameters influence solid–liquid interaction trends. Based on the binding energy analysis, the interaction mechanisms between solid bitumen and liquid hydrocarbon molecules across different maturation stages were systematically summarized.

**Table 1:** Ligand of saturates molecular docking.

Molecular Ligand	Molecular Structure	X_BP_
*n*–alkanes		
Ethane(C_2_H_6_)	H_3_C**—**CH_3_	0
……	……	……
*n*–gontane (C_10_H_22_)		0
……	……	……
*n*–triethane (C_30_H_26_)		0
*i*–hexadecane molecules		
Type I (C_16_H_34_)		0
Type II (C_16_H_34_)		0
Type V (C_16_H_34_)		0
Type VII (C_16_H_34_)		0
naphthenic molecules		
Trimethylene (C_3_H_6_)		0
……	……	……
Hexamethylene (C_6_H_12_)		0
……	……	……
Cyclohexane (C_10_H_20_)		0

**Table 2:** Ligand of aromatics molecular docking.

Molecular Ligand	Molecular Structure	Condensation mode	X_BP_
polycyclic aromatic hydrocarbons			
Benzene (C_6_H_6_)		/	0
Naphthalene (C_10_H_8_)		Conventionally linked	0.25
Anthracene (C_14_H_10_)		Conventionally linked	0.40
Pentaphenylbenzene (C_18_H_12_)		Conventionally	0.50
Pyrene (C_16_H_10_)		Polymerically linked	0.60
Pentabenzene (C_22_H_14_)		Conventionally	0.57
C_20_H_12_		Polymerically linked	0.67
Hexaflorobenzene (C_26_H_16_)		Conventionally	0.63
C_22_H_12_		Polymerically linked	0.83
Chloe (C_24_H_12_)		Ring linked	0.5
Heptaphenyls (C_30_H_18_)		Conventionally	0.67
C_26_H_14_		Polymerically linked	0.86
Naphthalene derivatives			
Methylnaphthalene		/	0.25
Propyl naphthalene		/	0.25
Pentyl naphthalene		/	0.25
Trimethyl naphthalene		/	0.25
Pentamethyl naphthalene		/	0.25

## Results

### Saturated hydrocarbons

#### Alkanes

**Fig. 2:**
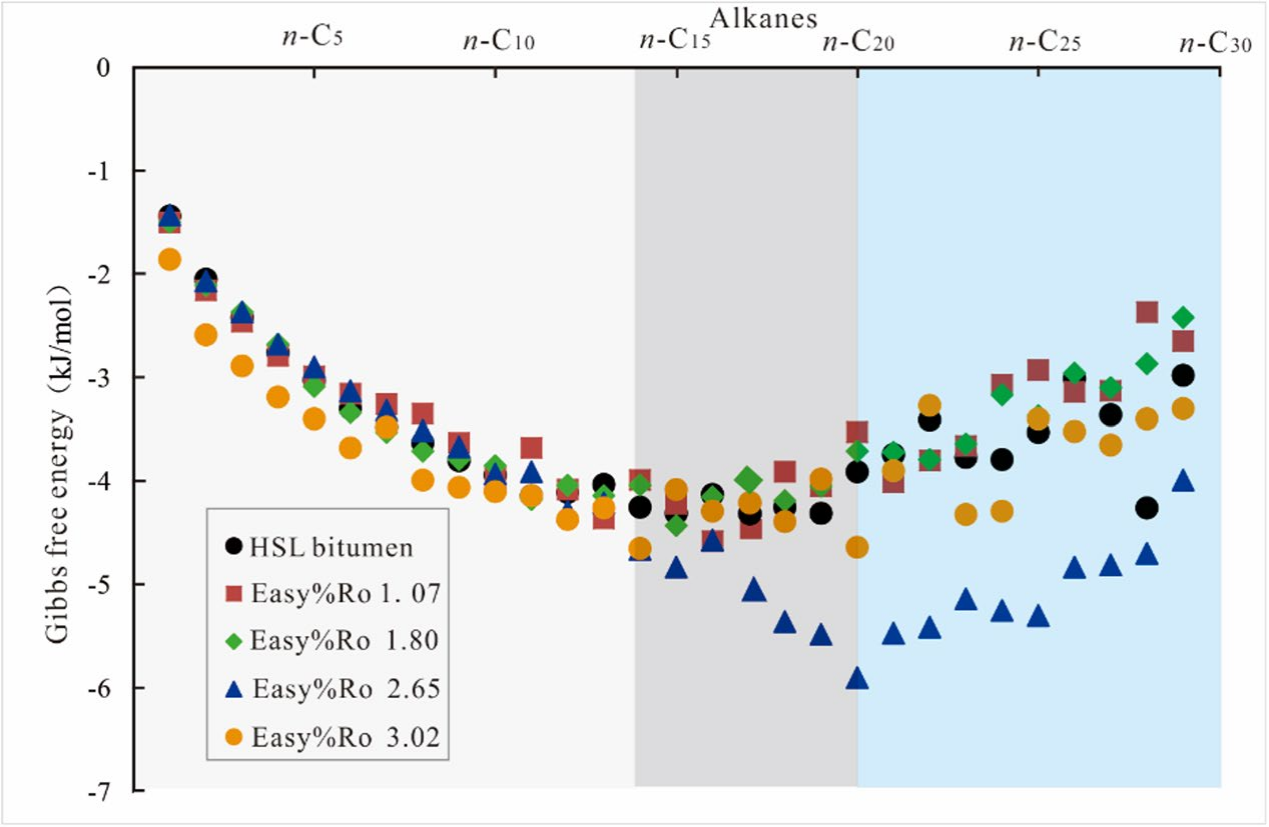
Changes in the binding energy between HSL solid bitumen and *n*–alkane.

Molecular docking calculations were performed between *n*–alkanes (C_2_–C_30_) and natural solid bitumen samples. The computational results are presented in Fig. 2. All calculated ΔG values for *n*–alkane binding to solid bitumen exhibit negative magnitudes, demonstrating thermodynamically favorable phase interactions^37^. This energetically driven phenomenon confirms the spontaneous adsorption capacity of solid bitumen for *n*–alkanes in the absence of external forces.

The binding energy between n–alkanes and solid bitumen exhibits three distinct distribution patterns. For C_2_–C_14_ *n*–alkanes, the binding affinity with solid bitumen strengthens progressively with increasing carbon number. C_15_–C_20_ *n*–alkanes exhibit consistently stronger binding affinities (ΔG values lower than − 4 kJ/mol) for solid bitumen. C_20_–C_30_ *n*–alkanes show a fluctuating upward trend in binding energy. C_2_–C_14_ *n*–alkanes, classified as light hydrocarbons, are relatively volatile. C_15_–C_20_ *n*–alkanes demonstrate higher stability compared to C_2_–C_14_ *n*–alkanes, while having shorter molecular chain lengths than C_20_–C_30_ *n*–alkanes. Consequently, solid bitumen structures preferentially enrich C_15_–C_20_ components among *n*–alkane compounds.

**Fig. 3:**
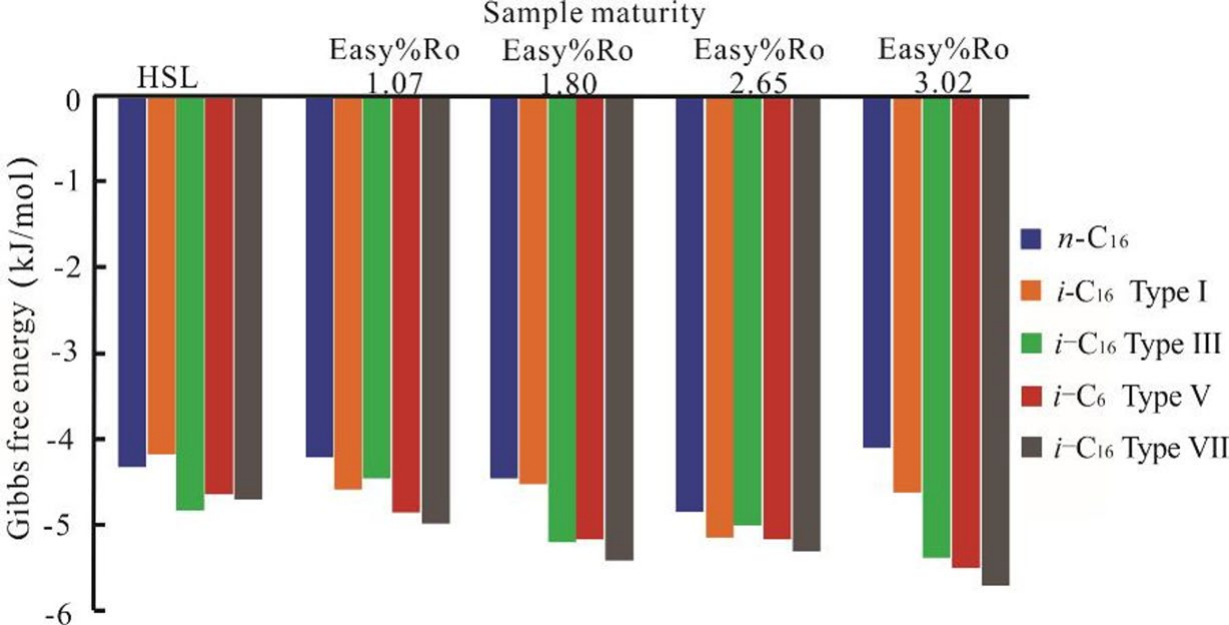
Changes in the binding energy between HSL solid bitumen and *i*–hexadecane molecules.

The molecular docking results between *i*–C_16_ (iso–hexadecane) and solid bitumen are illustrated in Fig. 3. The ΔG values required for iso–hexadecane binding to solid bitumen are systematically more negative than those of *n*–hexadecane, presumably due to the structural presence of additional methyl groups in the isomer.

### Cycloalkanes

**Fig. 4:**
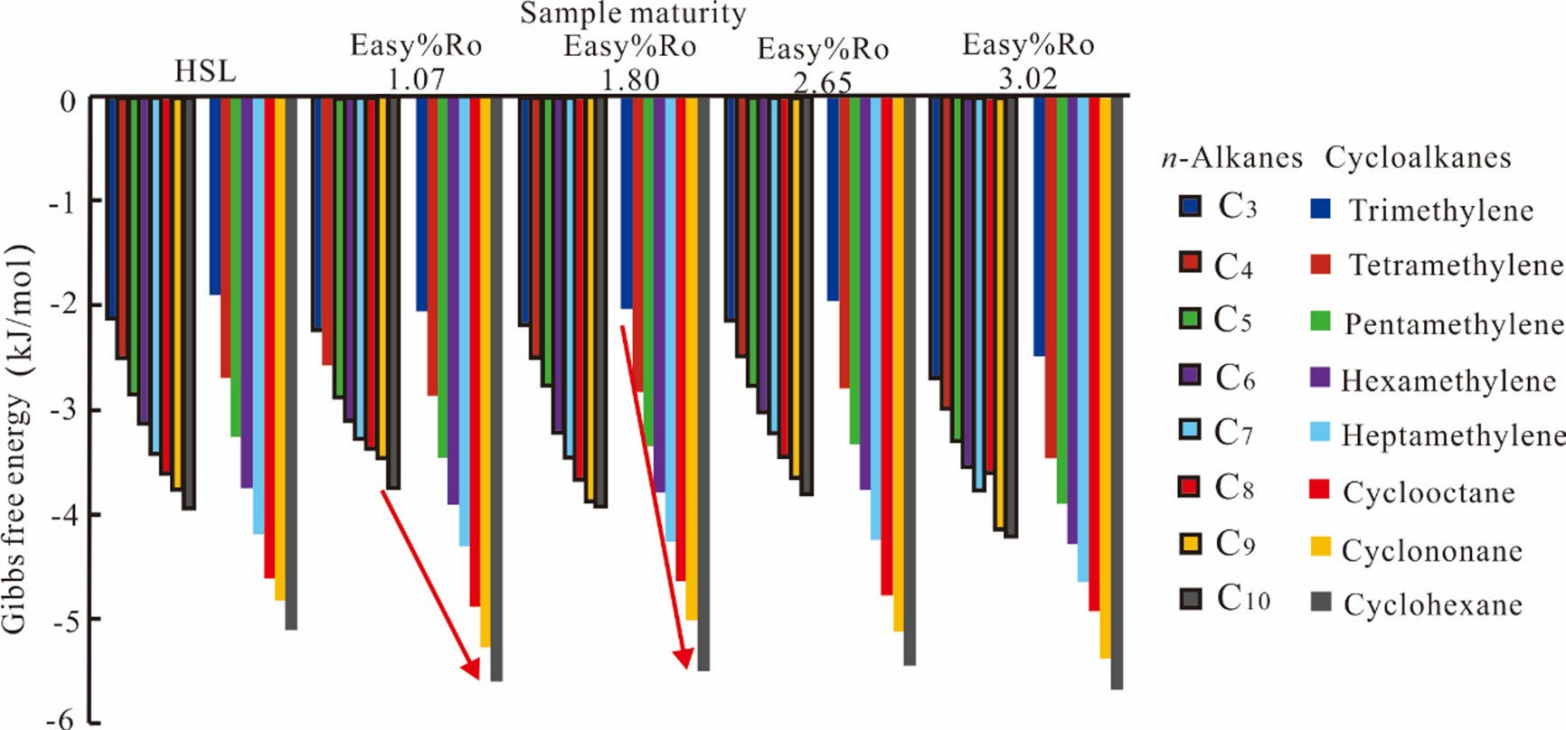
Changes in the binding energy between HSL solid bitumen and *n*–alkane, naphthenic molecules.

Cycloalkanes are saturated hydrocarbons characterized by an inherent degree of unsaturation in their cyclic molecular architectures. C_3_H_6_–C_10_H_20_ cycloalkanes constitute a common constituent of petroleum systems^38^. In this investigation, eight molecular ligands were selected, comprising cycloalkane compounds with sequentially incrementing carbon numbers. The computational analysis (Fig. 4) reveals that all eight cycloalkanes demonstrate negative ΔG values upon solid bitumen binding, characterized by a monotonic decrease in binding energy with increasing carbon chain length. These findings indicate that solid bitumen demonstrates preferential affinity for larger cycloalkane configurations. Comparative analysis with *n*–alkanes shows distinct behavior – thermodynamic stabilization of ΔG occurs only when carbon numbers surpass 14, following an initial progressive decline prior to reaching steady–state conditions. This comparative evidence suggests caution in extrapolating these findings to propose a systematic enhancement of cycloalkane–bitumen affinity with increasing molecular size.

For cycloalkanes and their alkane counterparts with equivalent carbon numbers, the binding energies of the cycloalkanes with solid bitumen are significantly reduced relative to their corresponding alkanes, except cyclopropane and propane. This phenomenon likely reflects that cycloalkanes with unsaturated structures are more readily adsorbed by solid bitumen than methyl–functionalized *n*–alkanes.

### Aromatic hydrocarbons

#### Benzene and polycyclic aromatic hydrocarbons

**Fig. 5:**
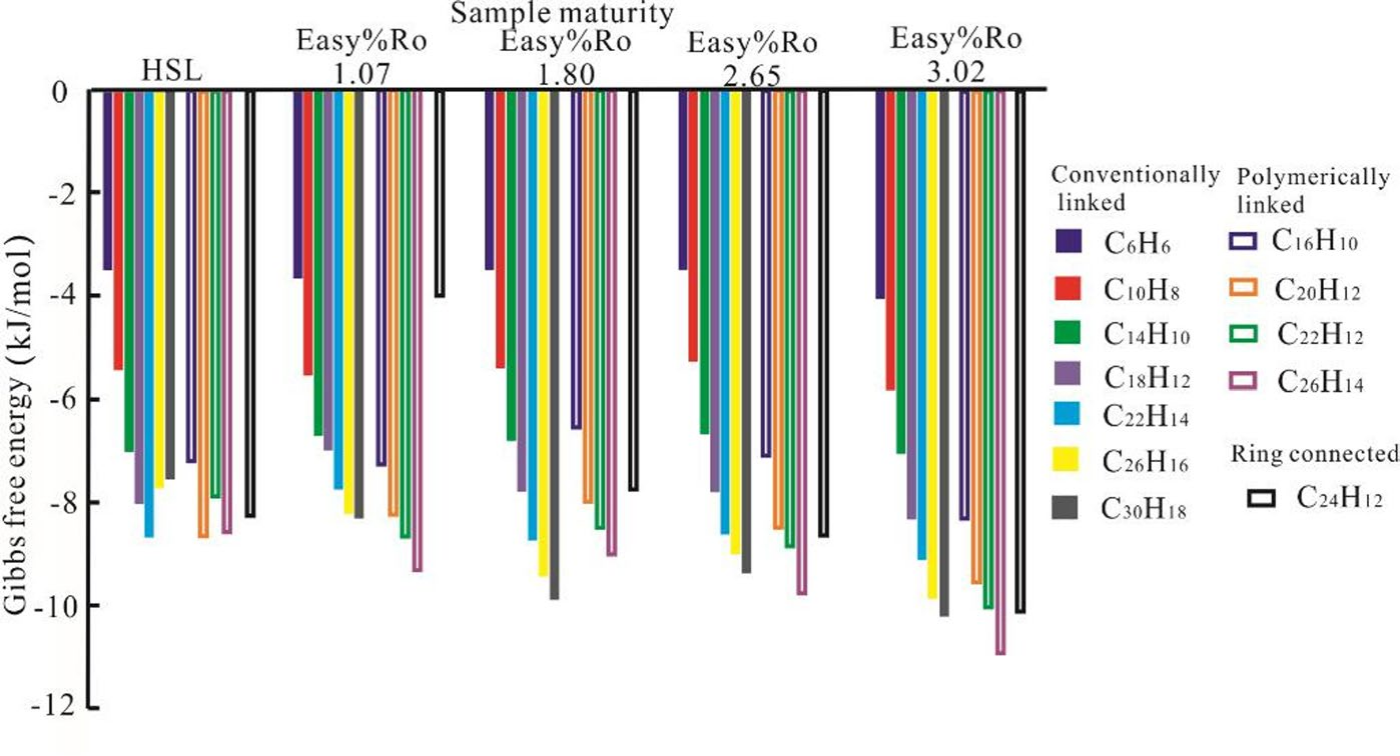
Changes in the binding energy between HSL solid bitumen and condensed aromatic hydrocarbons molecules.

In this study, benzene and PAHs were employed as molecular docking ligands. The PAHs include both linearly fused aromatic carbon clusters and cyclically fused aromatic carbon clusters^39^. Specifically, C_16_H_10_、C_20_H_12_、C_22_H_12_、C_26_H_14_ were designated as polymer–linked aromatic carbon clusters to investigate the binding characteristics of natural solid bitumen with PAHs sharing identical aromatic ring numbers but distinct molecular condensation degrees (X_BP_). X_BP_ serves as a key parameter characterizing the chemical structure of aromatic hydrocarbons, primarily reflecting the number of aromatic rings and their connectivity modes—such as linear, annularly fused, and polymeric linkages—which in turn determines the size and compactness of the aromatic system^40^. During the thermal cracking of crude oil, aromatic hydrocarbons undergo complex condensation reactions that lead to a gradual increase in their X_BP_. As a result, a strong correlation exists between the molecular condensation degree of aromatic compounds and the thermal maturity of crude oil. To investigate the effect of molecular condensation degree on solid–liquid adsorption, we performed molecular docking simulations using aromatic hydrocarbons with varying degrees of condensation. The ΔG results for molecular docking between benzene, PAHs, and solid bitumen of varying maturity levels are presented in Fig. 5.

When docking with the same solid bitumen, the binding energy of linearly fused PAHs increases progressively with carbon number, while polymer–linked PAHs exhibit the same trend. For PAHs with identical aromatic ring counts, the binding energy varies significantly depending on their linkage patterns. Specifically, among PAHs with an identical number of aromatic rings, the linearly fused isomers demonstrate the most favorable binding (most negative ΔG), whereas the polymer–linked and cyclically fused forms exhibit less favorable interactions (less negative ΔG) for binding to solid bitumen. This indicates that the molecular mass of aromatic hydrocarbons plays a determinative role in solid–liquid organic interactions. Under conditions of equivalent solid bitumen maturity, higher molecular mass of PAHs exhibit more favorable binding (as indicated by more negative ΔG values), which corresponds to a stronger interaction between solid and liquid organic phases.

### Polycyclic aromatic hydrocarbon derivatives

**Fig. 6:**
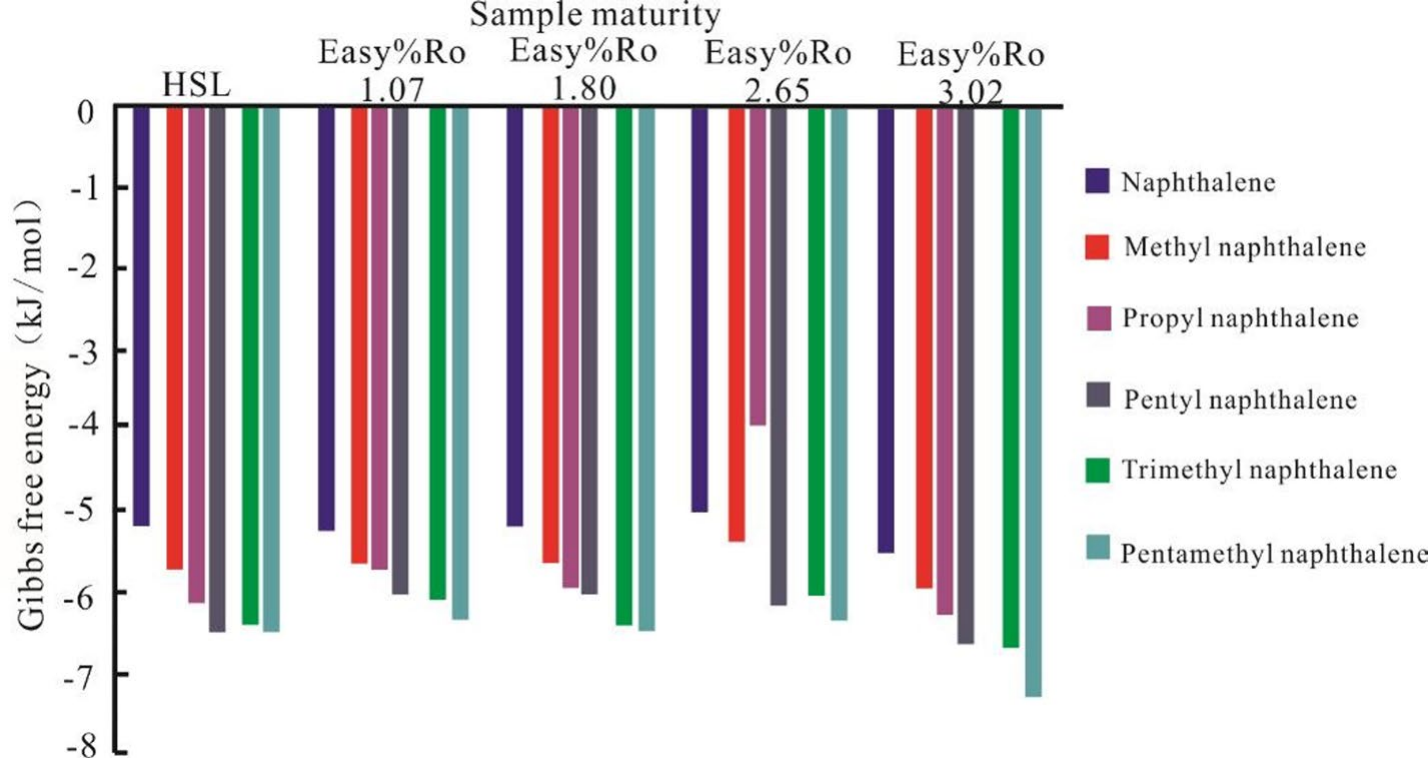
Changes in the binding energy between HSL solid bitumen and aromatic derivatives molecules.

In this study, the polycyclic aromatic hydrocarbon (PAH) derivatives employed include methylnaphthalene, *n*–propylnaphthalene, *n*–pentylnaphthalene, trimethylnaphthalene, and pentamethylnaphthalene. The results of molecular docking polycyclic aromatic hydrocarbon derivatives and solid bitumen are presented in Fig. 6.

Among aromatic hydrocarbons of equivalent number of aromatic rings, naphthalene exhibits the least favorable binding (higher ΔG) to solid bitumen. All alkyl–substituted naphthalene derivatives demonstrate stronger binding affinities than the parent naphthalene molecule. Among methylnaphthalene, *n*–propylnaphthalene, and *n*–pentylnaphthalene, the ΔG for binding to solid bitumen progressively decreases, indicating that *n*–pentylnaphthalene forms the tightest association with solid bitumen. Similarly, for polymethylnaphthalenes (methylnaphthalene, trimethylnaphthalene, and pentamethylnaphthalene), the ΔG values show a gradual decline with increasing methyl substitution. This demonstrates that, at equivalent condensation degrees the combined influence of methyl group quantity and molecular mass leads to the strongest binding affinity for pentamethylnaphthalene.

*n*–Propylnaphthalene and trimethylnaphthalene, *n*–pentylnaphthalene and pentamethylnaphthalene also share identical molecular masses. However, for the same solid bitumen sample, trimethylnaphthalene and pentamethylnaphthalene exhibit more negative ΔG values compared to *n*–propylnaphthalene and *n*–pentylnaphthalene, respectively. This indicates that methylene groups exert a minimal influence on solid–liquid interactions. When compounds possess equivalent molecular masses, the number of methyl substituents becomes the dominant factor governing organic binding affinity.

## Discussion

### Types of liquid hydrocarbon compounds

The ΔG values for the binding of C_2_–C_10_ n–alkanes and C_3_H_6_–C_10_H_20_ cycloalkanes to solid bitumen samples range from − 4.10 to − 1.18 kJ/mol and from − 5.70 to − 4.08 kJ/mol, respectively. For benzene, PAHs, and PAH derivatives, the corresponding ΔG ranges are − 10.35 to − 3.38 kJ/mol and − 7.35 to − 3.94 kJ/mol, respectively. Comparative analysis indicates that for linear alkanes and cycloalkanes with similar carbon numbers, the binding capacity of cycloalkanes to solid bitumen is significantly stronger than that of linear alkanes (Fig. 4). For aromatic hydrocarbons and saturated hydrocarbons with similar carbon numbers, solid bitumen demonstrates higher adsorption capacity for aromatic hydrocarbons. The adsorption capacity of solid bitumen for liquid hydrocarbons is influenced not only by the molecular weight of the hydrocarbons but also by their molecular structure type (e.g., *n*–alkanes, iso–alkanes, aromatic hydrocarbons). This finding aligns with the conclusions of Liang et al. (2024)^41^ and Li et al. (2023)^42^, yet contrasts with our initial hypothesis presented in the Introduction.

### The molecular mass

The computational docking results for *n*–alkanes, cycloalkanes, and polycyclic aromatic hydrocarbons (PAHs) with solid bitumen are presented in Figs. 4 and 5, respectively. The ΔG required for the binding of cycloalkanes and PAHs to solid bitumen decreases progressively with increasing molecular mass. As shown in Fig. 6, the ΔG for the binding of naphthalene, methylnaphthalene, *n*–propylnaphthalene, and *n*–pentylnaphthalene to the same solid bitumen gradually declines as the molecular mass of PAH derivatives increases. Molecular mass therefore serves as a critical determinant in solid–liquid interfacial interactions. The binding tightness between solid bitumen and most petroleum–derived cycloalkanes and PAHs exhibits a positive correlation with molecular mass. Meanwhile, no clear linear relationship was observed between the maturity of solid bitumen and its adsorption capacity for liquid hydrocarbons. This trend runs counter to our initial hypothesis outlined in the Introduction but aligns with the conclusions reported by Endo et al. (2008)^43^.

### Degree of molecular condensation

According to Figs. 2 and 5, for alkanes and PAHs with equal carbon numbers (e.g., C_14_ *n*–alkane vs. C_14_H_10_ PAH, C_26_ *n*–alkane vs. C_26_H_16_ PAH), although *n*–alkanes exhibit slightly higher molecular masses than PAH counterparts, PAHs demonstrate significantly stronger binding capability to solid bitumen. This indicates that the X_BP_ of compounds exerts a substantial influence on solid–liquid intermolecular interactions. The X_BP_ values for pentaphene (C_22_H_14_) and condensed polyaromatic hydrocarbon (C_22_H_12_) were determined as 0.6 and 0.83, respectively. Although C_22_H_14_ exhibits a marginally higher molecular weight compared to C_22_H_12_, its ΔG demonstrates greater elevation when bound to identical solid bitumen. A similar trend is observed in conventionally linked C_26_H_16_ and its polyaromatic derivative (C_26_H_14_). These comparative analyses indicate that PAHs with higher condensation degrees exhibit stronger binding affinity to solid bitumen molecules, which aligns with the findings reported by Handle et al. (2017)^44^. The condensation degree is a key factor not considered in our initial hypothesis.

As shown in Fig. 5, the ΔG exhibits a progressive decline with increasing degree of condensation for isostructural PAH configurations. However, when the structural configuration of the PAHs is altered, the polymerized PAH species exhibit a reduced binding affinity to solid bitumen compared to their conventionally bonded counterparts with equivalent ring counts. This phenomenon arises from the lower molecular weight of polymerically connected condensed aromatic compounds relative to conventional analogs with identical aromatic ring counts, coupled with the diminished binding capacity of highly condensed aromatic systems to solid bitumen compared to conventionally connected aromatic compounds.

### Number of Methyl groups

Figures 3 and 6 demonstrate the influence of methyl group content on solid–liquid organic molecular interactions. Comparative analysis of saturated hydrocarbons reveals that iso–alkanes with elevated methyl group concentrations display significantly stronger binding affinities to solid bitumen relative to the *n*–alkane counterparts at equivalent carbon numbers. This trend persists in aromatic systems, where structural isomers with comparable condensation degrees and molecular masses exhibit progressively enhanced binding capabilities to solid bitumen with increasing methyl group contents. The methyl groups in liquid hydrocarbons primarily influence solid–liquid interactions by enhancing the hydrophobicity and van der Waals forces of the hydrocarbon compounds (Kalpathy, 2015)^45^. Therefore, when investigating the adsorption mechanisms of hydrocarbons onto solid organic matter, careful attention should be paid to the role of methyl groups.

In summary, solid–liquid organic interactions are governed by hydrocarbon type, molecular mass, methyl group content, and degree of molecular condensation within the system. These parameters act in concert, exhibiting no discernible hierarchical prioritization. Therefore, during primary hydrocarbon expulsion of natural solid bitumen, the generated crude oil predominantly contains light hydrocarbons, small–molecular naphthenic and aromatic compounds. Subsequent secondary hydrocarbon expulsion, triggered by temperature and pressure variations, yields products characterized by diminished light hydrocarbon content, primarily releasing polycyclic aromatic hydrocarbons and methane derived from thermal cracking of polymethyl aromatic macromolecules. In addition, in order to enhance aromatic hydrocarbon recovery during oil and gas extraction processes, polymethyl polycyclic aromatic hydrocarbon polymers can be incorporated into injection chemical formulations. This mechanism operates through displacement interactions whereby the introduced polymers effectively release polycyclic aromatic hydrocarbon compounds adsorbed within solid bitumen.

## Conclusions

This study employs semi–flexible molecular docking simulations between three–dimensional molecular models of solid bitumen with varying maturity levels and different saturated and aromatic hydrocarbons to investigate the governing mechanisms of solid–liquid organic interactions. Statistical analysis of ΔG values revealed the following key findings:

(1) The chemical nature of organic compounds critically governs their solid–liquid interfacial interactions. C_15_–C_20_ *n*–alkanes and cycloalkanes demonstrate a higher propensity for liberation from solid bitumen. Notably, aromatic hydrocarbons generally exhibit superior binding capabilities to bituminous surfaces relative to most saturated hydrocarbons.

(2) Molecular mass serves as a key determinant governing organic solid–liquid intermolecular interaction. Higher molecular mass cycloalkanes and aromatic hydrocarbons demonstrate enhanced retention within solid bitumen.

(3) The degree of condensation critically regulates organic solid–liquid interfacial interactions. Aromatic hydrocarbons demonstrate enhanced binding affinities toward solid bitumen relative to saturated hydrocarbons with comparable molecular weights. In contrast, PAHs possessing higher condensation degrees exhibit reduced binding energies compared to conjugated aromatic hydrocarbons with linear configurations.

(4) The methyl group concentration within liquid hydrocarbon compounds serves as a critical determinant of organic molecular interactions at the solid–liquid interface. Solid bitumen demonstrates enhanced adsorption affinity toward hydrocarbon species containing elevated methyl substituents, irrespective of their classification as saturated or aromatic compounds.

## Data Availability

No datasets were generated or analysed during the current study.
